# Abnormal Flagging of Prostate Specific Antigen Screening Tests: A Regression Discontinuity Design

**DOI:** 10.1007/s11606-025-10075-x

**Published:** 2025-12-09

**Authors:** Patrick Lewicki, Ralph Jiang, Archana Radhakrishnan, Matthew Schipper, Todd Morgan, Kristian Stensland

**Affiliations:** 1 Department of Urology, University of Michigan, Ann Arbor, MI USA; 2 Department of Biostatistics, University of Michigan, Ann Arbor, MI USA; 3 Department of Internal Medicine, University of Michigan, Ann Arbor, MI USA; 4 Department of Radiation Oncology, University of Michigan, Ann Arbor, MI USA; 5 Department of Learning Health Sciences, University of Michigan, Ann Arbor, MI USA

## Abstract

**Background:**

There is no explicitly normal or abnormal level of prostate specific antigen (PSA) in the blood, yet the lab reported “upper limit of normal” (ULN) represents the most accessible guidance available to physicians interpreting these tests and is agnostic to risk factors and life expectancy. We hypothesize that “abnormal flagging” on “elevated” results is a potent driver of downstream care irrespective of a patient’s underlying prostate cancer risk.

**Methods:**

Regression discontinuity design was applied to a cohort of patients undergoing PSA screening at a single center to isolate and estimate the impact of “abnormal flagging” of results above the PSA ULN on subsequent early repeat (“confirmatory”) PSA testing, urology referral, and prostate MRI or biopsy (or both).

**Results:**

Amongst 38,042 screening PSA tests, 1041 (2.7%) were flagged as abnormal. Adjusting for patient age and PSA gap, “abnormal” flagging of PSA (versus “normal”) was associated with an increased odds of repeat PSA testing (OR: 14.17; 95% CI 11.53–17.43; *p* < 0.001), referral (odds ratio (OR): 10.03; 95% confidence interval (CI) 7.53–13.47; *p* < 0.001), and either prostate MRI or biopsy (or both; OR 13.61; 95% CI 8.81–21.64; *p* < 0.001).

**Conclusions:**

While the use of PSA ULN has recognized shortcomings, abnormal flagging of results above this threshold strongly influences additional testing, referral, and biopsy, potentially leading to risk-discordant over- and under-evaluation. An improved reporting standard may include risk estimates of meaningful endpoints such as the risk of prostate cancer metastasis or death.

**Supplementary Information:**

The online version contains supplementary material available at 10.1007/s11606-025-10075-x.

## INTRODUCTION

While an estimated 10 million prostate-specific antigen (PSA) tests are performed for prostate cancer (PCa) screening annually,^1^ authoritative guidance on results interpretation, subsequent referral, and downstream evaluation is lacking. There is no specific normal or abnormal serum PSA level, but results are typically dichotomized as such based on a lab-reported “reference range”.^2^ “Reference ranges” are problematic in the case of PSA for numerous reasons including outdated evidence,^3^ absent validation,^4^ modest performance characteristics,^2^ and agnosticism towards important risk factors such as family history, race, and life expectancy.^5^ While clinical trials have evaluated the benefit of PSA screening using referral algorithms with PSA cutoffs,^6^ no head-to-head prospective comparison has proven that a given cutoff is more appropriate than others.

Despite these limitations, dichotomized “abnormal flags” on PSA results represent the most available piece of advice to primary care physicians (PCPs) at the time of test interpretation, potentially dictating evaluation based on flawed cutoffs and leading to management that does not reflect underlying risk. Differences in downstream evaluation immediately adjacent to a PSA upper limit of normal (ULN, above which higher results are flagged “abnormal”) are risk-discordant, given that patients with nearly identical PSA have nearly identical risk, controlling for other factors. Importantly, health system-level policy, rather than the decision making of individual PCPs and urologists alike, is implicated in risk-discordant care as physicians are strongly motivated to act in the presence of a positive cancer screening result.

Here, we use regression discontinuity to isolate and estimate the impact of “abnormal flagging” (i.e., PSA results dichotomized by an arbitrary ULN) on PCa referral and subsequent work-up. Our objective is to demonstrate the potency of this construct (“abnormal flagging”) to influence downstream behavior, emphasizing the importance of a thoughtful consideration of how these thresholds are set in cancer screening. We also discuss the suitability of calculated risk estimates as triggers for “abnormal flagging” rather than unadjusted PSA results alone.

## METHODS

Regression discontinuity is a quasi-experimental design that attempts to estimate the impact (on, e.g., subsequent evaluation/management or clinical outcomes) of a cutoff or threshold-based policy that has been extrinsically applied to a continuous “running” variable.^7^ Here, PSA is the continuous variable of interest, the threshold-based policy is the abnormal flag applied above the PSA ULN, and the endpoint impacted or not impacted by the policy is additional prostate cancer-related evaluation. If the abnormal flag does not impact additional downstream evaluation, then the regression of PSA on additional evaluation endpoints should be continuous around the PSA ULN. Since biologic risk and confounding variables should be evenly distributed immediately above and below this cutoff, regression discontinuity allows us to isolate the effect of the abnormal flag on downstream evaluation. Further, since prostate cancer risk is practically identical immediately flanking a PSA ULN, discontinuity in subsequent work-up represents risk-discordant evaluation—potentially both under-evaluation and over-evaluation of those just below and above the ULN, respectively.

### Cohort

Serum PSA tests were queried from an institutional electronic medical record (EMR) data resource named DataDirect. DataDirect allows approved users to query health data entered into the EMR for more than 5 million patients at the University of Michigan. We defined “screening” as PSA testing in an asymptomatic patient for the purpose of detecting undiagnosed prostate cancer, excluding tests performed for follow-up of previously diagnosed prostate cancer or elevated PSA. By extension, diagnoses such as “urinary incontinence” were also exclusionary, considering that they are highly suggestive of previously treated prostate cancer, while other urologic conditions such as nephrolithiasis are not suggestive of a prostate cancer history and were therefore not exclusionary (Supplementary Table 1). Patients with lower urinary tract diagnoses that may obfuscate the interpretation of PSA were also excluded, along with PSA tests ordered by providers within Urology, Radiation Oncology, or Medical Oncology settings. This stringent approach favors specificity over sensitivity, creating a homogenous study cohort that may exclude some screening patients. The rationale and validity of this cohort have been described previously.^8^

Institutional guidance and local practice are important contexts for this work. Our institution is a large tertiary care center with a network of primary care offices throughout the state. Greater than 600 unique providers contributed PSA orders to the current dataset. The institution does not offer an independent recommendation on patient selection for prostate cancer screening, or how screening should be conducted. Prostate cancer screening is not a performance metric, nor are there any “best practice alerts” embedded in the EMR that may influence the use of a PSA test. Further, there are no formal requirements on who can or should be referred to a urologist, and there is no requirement for repeat testing or MRI prior to urologist referral. Within the department of urology, no formal protocols for the use of biomarkers, MRI, and biopsy exist.

### Endpoints and Covariates

Study endpoints captured downstream evaluation and management and included early repeat (potentially “confirmatory”) PSA testing, urology referral for PSA-related diagnosis, and receipt of either prostate MRI or biopsy (or both). All endpoints were measured within 6 months of a given PSA result. MRI and biopsy were analyzed as a combined endpoint, given that they can be competing diagnostic tests (i.e., the result of one may obviate the need for the other). Referral to urology for non-PSA-related diagnoses did not constitute a met endpoint (e.g., if a patient was referred to a urologist with a referral diagnosis of nephrolithiasis or erectile dysfunction, this was not considered to be a referral triggered by a PSA test result).

The primary covariate of interest, serving as the continuous “running” variable was “PSA gap.” Given that the PSA ULN varies by age at our institution (Table 1), PSA gap, by measuring the “PSA distance” between a given test result and age-adjusted ULN, allows us to classify patients of different ages on the same PSA scale. The PSA gap is not directly calculated by our institutional EMR, but for this analysis, it permits comparison of patients in differing age groups (since PSA ULN varies by age). A positive PSA gap represents an “abnormal” lab test and is flagged as such in the EMR. For example, a 55-year-old male with a PSA value of 3.2 ng/mL would have a PSA gap of (3.2–3.5) = −0.3 and would be labelled “normal.” A 55-year-old male with a PSA value of 4.2 ng/mL would have a PSA gap of (4.2–3.5) =  + 0.7 and would be flagged “abnormal.” A 65-year-old with a PSA value of 5.2 ng/mL would have a PSA gap of (5.2–4.5) =  + 0.7 and would be flagged “abnormal.”

**Table 1:** Institutional Age-specific PSA Normal Range. Above the Upper Limit of Normal, Results Are Flagged as “Abnormal”

Age (years)	PSA (ng/mL)
40–49	0–2.5
50–59	0–3.5
60–69	0–4.5
≥70	0–6.5

As a sensitivity analysis, we fit similar regression discontinuity models by age subgroups (50–59 and 60–69 years old, with “abnormal” thresholds of 3.5 and 4.5 ng/mL, respectively; Table 1) using actual PSA values as the continuous variable of interest rather than “PSA gap.” Given that practice patterns around downstream testing such as MRI and biopsy likely changed during our study period, a further sensitivity analysis applied the same regression discontinuity approach to PSA gap on receipt of MRI or biopsy (or both), stratifying by year of PSA test (July 2015–June 2019, July 2019–July 2023).

Additional covariates included history of prostate cancer in a first-degree relative, patient-reported race, and patient age.

### Analysis

Continuous and categorical variables were summarized as median (interquartile range (IQR)) and proportion, respectively.

Discontinuity at the PSA ULN (“normal”/”abnormal” cutoff) was estimated via logistic regression (Supplement).^9^ A statistically significant term for (PSA gap > 0) rejects the null hypothesis of no discontinuity at the PSA ULN and suggests an influence of the “abnormal flag” on downstream clinical endpoints. Age was included as a term in this regression given its association with PSA level and a suspected association with study endpoints.

Regression discontinuity design assumes that all potentially relevant variables besides the threshold policy variable (here, abnormal flagging above the ULN) are continuous across the threshold, and tests to what extent the endpoint variable (urologist referral, repeat PSA, MRI/biopsy) is continuous or discontinuous. To address this assumption, patient age, self-reported race, family history, and PSA level were entered into both the Prostate Biopsy Collaborative Group (PBCG) calculator and Prostate Cancer Prevention Trial (PCPT) calculator to estimate each patient’s risk of clinically significant PCa diagnosis on biopsy, serving as a composite baseline characteristic on which to assess continuity.^10,11^ While these calculators are not validated for the studied endpoints (i.e., they do not attempt to predict “need for referral”), their purpose is to test whether prostate cancer risk is continuous across the ULN threshold. Further, regression discontinuity assumes that assignment around the threshold is random, and that, for example, patients just below the ULN are not cautiously “up-graded” to “abnormal” by a laboratory. To address this assumption, we plot the distribution of patients relative to the ULN as a type of density test.^12^

Statistics were performed in R (version 4.3.0, R Foundation, Inc.). This study was exempted from institutional review board review (HUM#00245443).

## RESULTS

A total 38,042 PSA tests were performed on 17,734 patients from July 2015 to July 2023. Median (IQR) PSA was 1.0 ng/mL (0.6–1.7 ng/mL), with 1041 (2.7%) tests flagged as abnormal, 1648 (4.3%) tests followed by an early repeat/confirmatory test, 712 (1.9%) followed by a urology referral (Table 2), and 9340 (24.6%) within ULN ± 2 ng/mL. Median (IQR) PSA amongst flagged results was 5.1 ng/dL (4.3–6.9 ng/dL); 550 (53%) such tests were followed by repeat/confirmatory PSA, 538 (52%) by referral to a urologist, and 260 (25%) by MRI or biopsy (or both) within 6 months.

**Table 2:** Study Cohort Characteristics by (a) Patient-level and (b) Individual PSA Test-level. Number of Observations (Percent of Cohort) and Median (interquartile Range) are Shown for Categorical and Continuous Variables, Respectively

(a)	
Patients undergoing PSA testing	*N* = 17,734^*1*^
Self-identified African-American	1566 (8.8%)
First degree relative with prostate cancer	2459 (14%)
(b)	
Serum PSA tests	*N* = 38,042^*1*^
Value	1.00 ng/mL (0.60, 1.70)
PSA gap (PSA value minus ULN)	−2.80 ng/mL (−3.50, −2.00)
Flagged “abnormal”	1041 (2.7%)
Patient age at PSA test	59 years (53, 65)
Referred to urology within 6 months	712 (1.9%)
Repeat PSA within 6 months	1648 (4.3%)
Prostate MRI within 6 months	216 (0.6%)
Prostate biopsy within 6 months	246 (0.6%)

**Figure 1:**
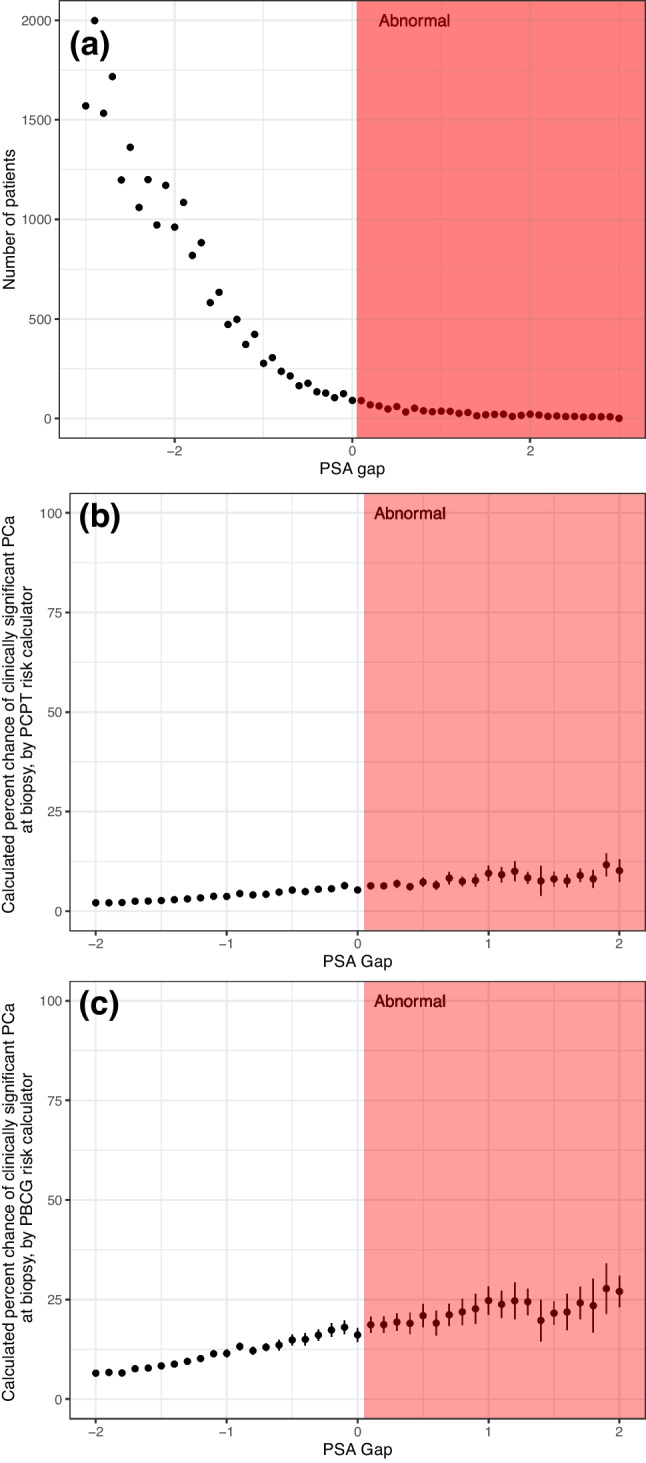
Graphical tests of the continuity assumption of regression discontinuity design. (**a**) Number of patients with a given “PSA gap;” (b-c) mean (and 95% confidence interval) calculated risk of clinically significant prostate cancer on biopsy via Prostate Cancer Prevention Trial (**b**) and Prostate Biopsy Collaborative Group (**c**) risk calculators. Pink shading corresponds to PSA tests labelled as “abnormal.”

Figures represent the 9340 (24.6%) test results that fell within ULN ± 2 ng/mL. Graphical tests of regression discontinuity assumptions are shown in Fig. 1. Distribution of patients as well as baseline characteristics (summarized as PBCG and PCPT calculator estimates) is continuous across the PSA ULN. Baseline characteristics are also individually shown to be continuous in Supplementary Fig. 1.

**Figure 2:**
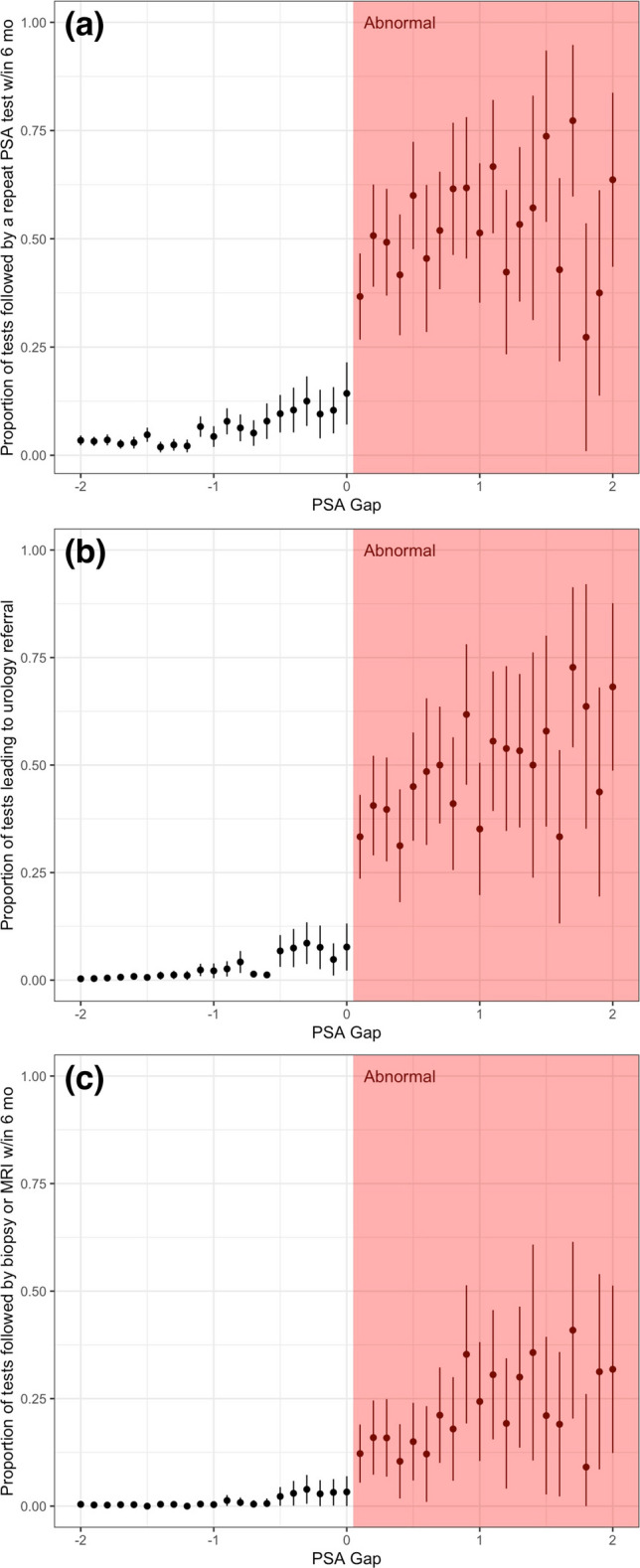
Proportion of PSA tests followed by (**a**) referral to urologist within 6 months, (**b**) repeat PSA test within 6 months, and (**c**) receipt of either prostate MRI or prostate biopsy (or both) within 6 months; by PSA gap. Error bars represent 95% confidence interval. Pink shading corresponds to PSA tests labelled as “abnormal.”

Figure 2 shows endpoints as a function of PSA gap. Results of a multivariable model assessing regression discontinuity are shown in Table 3. Adjusting for patient age and PSA gap, “abnormal” flagging of PSA (versus “normal”) was associated with an increased odds of early repeat/confirmatory PSA testing (OR 14.17; 95% CI 11.53–17.43; *p* < 0.001), referral (odds ratio (OR) 10.02; 95% confidence interval (CI) 7.53–13.47; *p* < 0.001), and either prostate MRI or biopsy (or both; OR 13.61; 95% CI 8.81–21.64; *p* < 0.001).

**Table 3:** Multivariable Models Assessing Regression Discontinuity of (a) Referral, (b) Early Repeat PSA, and (c) MRI or Biopsy (or both) at the PSA ULN

(a)				
	Odds ratio	95% CI lower bound	95% CI upper bound	*P*-value
Age (per 1 year)	1.05	1.04	1.06	< 0.001
PSA gap	4.23	3.62	4.96	< 0.001
**PSA gap > 0**	**10.03**	**7.53**	**13.47**	** < 0.001**
Interaction of PSA gap with PSA gap > 0	0.24	0.20	0.28	< 0.001
(b)				
	Odds ratio	95% CI lower bound	95% CI upper bound	*P*-value
Age (per 1 year)	1.05	1.04	1.06	< 0.001
PSA gap	1.39	1.32	1.47	< 0.001
**PSA gap > 0**	**14.17**	**11.53**	**17.43**	** < 0.001**
Interaction of PSA gap with PSA gap > 0	0.74	0.69	0.78	< 0.001
(c)				
	Odds ratio	95% CI lower bound	95% CI upper bound	*P*-value
Age (per 1 year)	1.02	1.00	1.03	0.015
PSA gap	2.66	2.17	3.27	< 0.001
**PSA gap > 0**	**13.62**	**8.81**	**21.64**	** < 0.001**
Interaction of PSA gap with PSA gap > 0	0.38	0.31	0.47	< 0.001

Sensitivity analyses grouping patients by age (50–59 and 60–69 years old) and using actual PSA as the continuous variable (with “abnormal” thresholds of 3.5 and 4.5 ng/mL for 50–59 and 60–69, respectively; Table 1) rather than calculated PSA gap yielded similar results (Supplementary Figs. 2 and 3; Supplementary Tables 2 and 3), except for the early repeat PSA endpoint amongst 60–69-year-olds, which was not statistically significantly associated with abnormal flagging. A final sensitivity analysis stratifying patients by date of PSA test and probing the possible influence of changes over time in the downstream diagnostic pathway (e.g., use of MRI prior to biopsy) demonstrated similar results across both groups (Supplementary Table 4), though the odds ratio was smaller amongst more recent PSA tests.

## DISCUSSION

In light of the continuous prostate cancer risk represented in Fig. 1b and c, discontinuity and dichotomy in referral and downstream management of patients associated with the presence of an “abnormal flag” are risk-discordant. Risk is continuous and similar near PSA thresholds, while evaluation is not. As such, non-evidence-based lab cutoffs appear to impact referral, additional testing, and biopsy, and potentially leading to both over- and under-evaluation amongst patients flanking the reported ULN. Two patients with identical risk may undergo widely divergent subsequent evaluation, including receipt of invasive testing, because of “abnormal flagging.” Our results highlight the potency of this construct and emphasize the importance of careful design of results reporting in cancer screening given that physicians are generally expected to and do act in response to an “abnormal” screen. As seen in Fig. 2, the slightly diminished rate of referral compared to repeat/confirmatory testing and of MRI/biopsy compared to referral suggests that physician-level discretion does enter into these decisions and that some repeat/confirmatory tests were below the ULN. Nonetheless, given the scale of PSA testing, this unintended consequence and the benefits of its remediation could apply to a large number of patients each year.^1^ The large behavioral effect of the “abnormal flag,” a readily modifiable component of laboratory and electronic medical record systems, also emphasizes an opportunity for improved communication around a patient’s underlying prostate cancer risk.

Challenges with laboratory “normal” or “reference” ranges are well recognized.^13,14^ Reference ranges may not represent the statistical distribution of values of healthy individuals (which may be skewed, bimodal, etc.); they typically report the central 95th percentile (a convenience cutoff based on standard deviation), and may not reflect clinically useful “decision limits” that set a threshold based on a desired sensitivity and specificity.^13^

While most lab tests are subject to regression discontinuity of downstream decision making at the boundary of a reference range, the demonstrated discontinuity in prostate cancer early detection is particularly troublesome in light of several considerations. For one, PSA in isolation performs modestly in discriminating amongst patients with and without prostate cancer due to confounding conditions such as BPH.^2^

Further, institutions reporting a single PSA reference range overlook the fact that PSA increases with age, exacerbating observed risk-discordant management by encouraging evaluation of older patients with shorter life-expectancy.^5^ While survival benefit has been demonstrated in the clinical trial setting via a single cutoff (≥ 3.0 ng/mL across most centers in the European Randomized study of Screening for Prostate Cancer (ERSPC)^15^), age- or risk-based cutoffs may be positioned to maximize benefits while also addressing overevaluation, overdiagnosis, and overtreatment. Our health system uses age-based cutoffs, which mitigates this effect, although ranges reported herein are based on an outdated cohort (from Oesterling et al., using Olmsted County data from the 1990s, early PSA screening adoption) and methods (range of PSA amongst patients without a digital rectal exam abnormality).^3^ Other work from this era, such as Morgan et al.’s study of PSA values amongst Caucasian and African-American patients with and without prostate cancer, similarly relies on outdated diagnostics and for this reason (along with the challenge of accounting for clinical stage and stage migration over the past three decades) is difficult to map to a modern cohort.^16^ Existing reference ranges or decision limits are based on data from the early era of PSA adoption, where they were favored for the predictive value,^17^ and were likely crystallized in large trials (Prostate, Lung, Colorectal and Ovarian Cancer Screening Trial using a threshold of ≥ 4.0 ng/mL,^18^ ERSPC using ≥ 3.0 ng/mL^6^) but face similar challenges given the changing nature of prostate cancer diagnosis over time^19^ and an evolved focus on clinically significant disease.^20^ Contemporary age-based cutoffs exist but are not widely circulated.

We believe that reporting calculated risk incorporating risk factors and life expectancy (with thresholds, if necessary) to be superior to flagging lab results in isolation both for decision making and patient counseling. Several candidate risk calculators integrating age, PSA, race, and family history exist for the prediction of clinically significant prostate cancer on biopsy.^10,11^ We envision in-line reporting (minimizing additional workload) and potentially multi-level threshold setting (e.g., urgent referral, referral, repeat annual screening, screening discontinuation) based on a validated risk calculation. Existing calculators do not adjust for the competing risk of other cause mortality, and therefore our future work will focus on developing a prostate cancer specific mortality risk calculator for this express purpose, to be used by PCPs and urologists alike in the early phase of downstream evaluation, derived from prospective clinical trial data with long follow-up and adjudicated cause of death and validated in contemporary, representative populations. While risk of positive biopsy may be informative, the clinical significance of biopsy pathology is dependent on biopsy strategy (i.e., template biopsy and imaging targeted biopsy), which varies historically and amongst providers.^21^ Further, decisions around surveillance versus treatment (and the consequent changes in health utilities) are informed by more than merely whether a biopsy reveals clinically significant prostate cancer.^22^ A user centered design process including patients and physicians will be used to optimize which risk estimates (positive biopsy, metastasis, prostate cancer-specific mortality) are reported and how they are reported. We believe that such application of risk-based, rather than PSA-based, thresholds could optimize recent clinical trial based sequential screening protocols, such as those involving 4K^23^ score and MRI.^24^ Nonetheless, this approach will still be subject to possible over- and under-diagnosis given that PSA drives most of the risk calculation and is still subject to the limitations of any diagnostic test.^25,26^ Decision thresholds based on risk estimates should reflect prior methodology studying PSA’s predictive value, rather than being based on statistical distribution alone.

Our work has several limitations. First, this is a single institution study, and results may not generalize to other settings, though the observed effect is likely more harmful still in institutions reporting a single, non-age adjusted reference range. Certain health systems may provide more comprehensive guidance on managing PSA results. As this EMR data resource is not linked to an institutional health plan, care received outside of our institution is not captured. The impact of missing longitudinal data is mitigated by studying each patient over a relatively short period of time (6 months). Second, we rely largely on structured data such as diagnosis and billing codes, which lack some clinical nuance. Relatedly, capture of family history may be incomplete in light of known documentation gaps,^27^ though positive family history was continuous across the PSA ULN (Supplement). Third, stringent exclusion criteria may diminish the number of real-world patients to whom these findings apply. Finally, different approaches to regression discontinuity modelling may result in different estimated odds ratios of the effect of the “flag,” and the actual magnitude of the odds ratio should not be overinterpreted.

That considered, we demonstrate a significant discontinuity in receipt of additional diagnostic evaluation at the lab reported PSA ULN, including not just referrals and labs but also invasive diagnostic tests such as prostate biopsy. This supports the notion that the “abnormal flag” functions as a strong heuristic for physicians evaluating PCa screening patients. PCPs and urologists alike behave expectedly and appropriately cautiously given an abnormal result—the onus is on institutions or organizations to critically evaluate how such results are presented and determined to be “abnormal.” Comprehensive risk estimates may be superior for shared decision making, and future work will involve patients, physicians, and leadership alike in a user-centered design process to this end.

## Supplementary Information

Below is the link to the electronic supplementary material.

Supplementary Material 1 (DOCX 1.30 MB)

## Data Availability

Data are not available to other researchers because they are from an institutional database of patients providing routinely collected data, although a limited dataset could be provided on reasonable request.
