# Supplementary material for: Abnormal Flagging of Prostate Specific Antigen Screening Tests: A Regression Discontinuity Design
Source: J Gen Intern Med. 2025 Dec 9;41(7):1743–9. doi: 10.1007/s11606-025-10075-x (PMC13176384; doi:10.1007/s11606-025-10075-x)
Supplement: Supplementary file 1 — Supplementary Material 1 (DOCX 1.30 MB) [file 11606_2025_10075_MOESM1_ESM.docx]

Supplement

**Cohort**

Cohort creation started with a definition of “prostate specific antigen (PSA) screening” as:

*PSA testing performed in an asymptomatic patient for the purpose of detecting undiagnosed prostate cancer.*

This definition excludes PSA testing that is performed after prostate cancer diagnosis or in patients with a previous history of elevated PSA (PSA testing in that context is for follow-up of an abnormal result, not for screening). Patients with lower urinary tract diagnoses that may obfuscate interpretation of PSA were also excluded. Finally, diagnoses that are highly suggestive of prostate cancer or treated prostate cancer in a patient undergoing PSA testing (e.g., urinary incontinence, history of radiation) were excluded. To enrich the cohort for PSA screening patients (recognizing that lists of pre-existing diagnoses may be incomplete for a variety of reasons), PSAs were only included if ordered by providers outside of Urology, Radiation Oncology, or Medical Oncology settings.

Structured medical history data in EMR was used to exclude PSA tests that were performed in patients with the below diagnoses *before* the time of PSA testing. Therefore, in the case of a patient with two PSA tests and an excluding diagnosis chronologically between them, only the PSA test prior to the excluding diagnosis would be included in the study cohort.

The list of excluding and endpoint codes was manually created from frequency tables of all patients undergoing PSA testing at our institution. Endpoint codes were PSA related and constituted a “referral endpoint” when combined with an order for referral or actual urologist visit.

**Supplementary Table 1.** Exclusion and Inclusion diagnoses

| \| **Exclusion: "Diagnosis Everything" table** \| \| --- \| \| Malignant neoplasm of prostate \| \| Neoplasm, Malignant, prostate \| \| Secondary malignant neoplasm of bone \| \| Malignant (primary) neoplasm, unspecified \| \| Personal history of malignant neoplasm of prostate \| \| Enlarged prostate without lower urinary tract symptoms \| \| Encounter for follow-up examination after completed treatment for malignant neoplasm \| \| Enlarged prostate with lower urinary tract symptoms \| \| Hyprtrphy prst bng w/o urinary obst \| \| Neop, mlig, disseminated \| \| Hx, prsnl, prostatic malignancy \| \| Secondary and unspecified malignant neoplasm of intrapelvic lymph nodes \| \| Retention of urine, unspecified \| \| Malignant neoplasm of bladder, unspecified \| \| Urinary tract infection, site not specified \| \| Rising PSA following treatment for malignant neoplasm of prostate \| \| Infection, urinary tract NOS \| \| Other obstructive and reflux uropathy \| \| Neop, mlig, bladder NOS \| \| Symptoms, other symptoms, urinary \| \| Symptom, retention, urine NOS \| \| Neop, mlig, lymph intrapelvic \| \| Erectile dysfunction following radical prostatectomy \| \| Irradiation cystitis without hematuria \| \| Hormone resistant malignancy status \| \| Unspecified urinary incontinence \| \| Neuromuscular dysfunction of bladder, unspecified \| \| Obstruction, urinary NEC \| \| Malignant neoplasm of overlapping sites of bladder \| \| Hyprtrphy prst bng w/urinary obst \| \| Urge incontinence \| \| Other retention of urine \| \| Symptom, incontinence, urinary NOS \| \| Bladder-neck obstruction \| \| Overactive bladder \| \| Neurogenic bladder NOS \| \| Obstruction, bladder neck \| \| Hyprplas, prst NOS w/urinary obst \| \| Erectile dysfunction following radiation therapy \| \| Cystitis, irradiation \| \| Nodular prostate without lower urinary tract symptoms \| \| Inflammatory disease of prostate, unspecified \| \| Other specified disorders of bladder \| \| Benign neoplasm of prostate \| \| Hypertonicity, bladder \| \| Cystitis, unspecified without hematuria \| \| Personal history of malignant neoplasm of bladder \| \| Acute cystitis without hematuria \| \| Carcinoma in situ of prostate \| \| CA in situ, prostate \| \| Obstruction, urinary NOS \| \| Neop, mlig, bladder NEC \| \| Prostatitis NOS \| \| Cystitis NOS \| \| Prostatic intraepithelial neoplasia \| \| Hyprplas lclzd bng w/urinary obst \| \| Calculus in bladder \| \| Nodular prostate w/o urinary obst \| \| Other specified disorders of prostate \| \| Atypical small acinar of prostate \| \| Other specified disorders bladder \| \| Cystitis, acute \| \| Personal history of urinary (tract) infections \| \| Interstitial cystitis (chronic) without hematuria \| \| Calculus in bladder NEC \| \| Dysplasia, prostate \| \| Cystostomy status NEC \| \| Irradiation cystitis with hematuria \| \| Hx, urinary infection \| \| Hx, prsnl, bladder malignancy \| \| Neoplasm of unspecified behavior of bladder \| \| Neop, UB, prostate \| \| Acute cystitis with hematuria \| \| Symptom, retention, urine NEC \| \| Hyprplas, prst NOS w/o urinary obst \| \| Acute prostatitis \| \| Malignant neoplasm of lateral wall of bladder \| \| Infection and inflammatory reaction due to indwelling urethral catheter, initial encounter \| \| Bladder disorder, unspecified \| \| Malignant neoplasm of posterior wall of bladder \| \| Disorder, prostate NEC \| \| Cystitis, chronic interstitial \| \| Chronic prostatitis \| \| Neop, NOS, bladder \| \| Disorder, bladder NOS \| \| Symptom, incontinence, urinary \| \| Prostatitis, acute \| \| Carcinoma in situ of bladder \| \| Symp incomplete bladder emptying \| \| Malignant neoplasm of dome of bladder \| \| Neop, mlig, bladder lateral \| \| CA in situ, bladder \| \| Functional urinary incontinence \| \| Prostatitis, chronic \| \| Malignant neoplasm of anterior wall of bladder \| \| Attention to urinostomy NEC \| \| Disorder, prostate NOS \| \| Symptom, leakage, continuous urine \| \| Other specified urinary incontinence \| \| Urinostomy status NEC \| \| Infct d/t indwelling urine catheter \| \| Benign neoplasm of bladder \| \| Flaccid neuropathic bladder, not elsewhere classified \| \| Calculus of prostate \| \| Neop, mlig, bladder posterior \| \| Disorder of prostate, unspecified \| \| Neop, mtstc, urinary NEC \| \| Abscess of prostate \| \| Neoplasm of uncertain behavior of bladder \| \| Fit/adjust urinary device \| \| Neop, mlig, bladder anterior \| \| Cystitis, unspecified with hematuria \| \| Malignant neoplasm of bladder neck \| \| Overflow incontinence \| \| Atony, bladder \| \| Symptom, incontinence, urinary NEC \| \| Secondary malignant neoplasm of bladder \| \| Other congenital malformations of bladder and urethra \| \| Other neuromuscular dysfunction of bladder \| \| Erectile dysfunction following simple prostatectomy \| \| Infection and inflammatory reaction due to other urinary catheter, initial encounter \| \| Disorder, urethra/urinary tract NOS \| \| Calculus, prostate \| \| Urinary catheterization as the cause of abnormal reaction of the patient, or of later complication, without mention of misadventure at the time of the procedure \| \| Nodular prostate with lower urinary tract symptoms \| \| Infect,oth extrnl stoma,urinary trc \| \| Other complication of other external stoma of urinary tract \| \| Nodular prostate w/ urinary obst \| \| Malignant neoplasm of trigone of bladder \| \| Calculus of lower urinary tract, unspecified \| \| Prostatodynia syndrome \| \| Hx of urinary malignancy NEC \| \| Neop, bng, prostate \| \| Neop, UB, bladder \| \| Synd, cauda equina w/neuro bladder \| \| Abscess, prostate \| \| Dysfunction, bladder NEC \| \| Disease, prostatic inflammatory NEC \| \| Stenosis of incontinent stoma of urinary tract \| \| Encounter for screening for malignant neoplasm of bladder \| \| Other lower urinary tract calculus \| \| Other chronic cystitis without hematuria \| \| Diverticulum of bladder \| \| Erectile dysfunction following prostate ablative therapy \| \| Infection and inflammatory reaction due to implanted urinary sphincter, initial encounter \| \| Compliance, low bladder \| \| Other chronic cystitis with hematuria \| \| Other cystitis with hematuria \| \| Other cystostomy complication \| \| Breakdown (mechanical) of urinary sphincter implant, initial encounter \| \| Breakdown (mechanical) of urinary sphincter implant, subsequent encounter \| \| Other mechanical complication of urinary sphincter implant, initial encounter \| \| Infection and inflammatory reaction due to indwelling urethral catheter, sequela \| \| Neop, mlig, bladder trigone \| \| Neop, bng, bladder \| \| Calculus in lower urinary tract NEC \| \| Hyprplas lclzd bng w/o urinary obst \| \| Neoplasm of uncertain behavior of prostate \| \| Congestion and hemorrhage of prostate \| \| Bladder replacement NEC \| \| Personal history of prostatic dysplasia \| \| **Exclusion: "Clarity Medical History" table** \| \| Prostate cancer (CMS/HCC) \| \| BPH (benign prostatic hyperplasia) \| \| Radiation therapy complication \| \| Prostate enlargement \| \| Bladder cancer (CMS/HCC) \| \| Urinary incontinence \| \| Prostate CA (CMS/HCC) \| \| Urinary retention \| \| Enlarged prostate \| \| Prostatic adenocarcinoma (CMS/HCC) \| \| History of prostate cancer \| \| Neurogenic bladder \| \| Carcinoma in situ of prostate \| \| History of radiation therapy \| \| Prostatitis \| \| Malignant neoplasm of prostate (CMS/HCC) \| \| BPH (benign prostatic hypertrophy) \| \| Prostate cancer metastatic to bone (CMS/HCC) \| \| Bladder neck contracture \| \| Benign localized prostatic hyperplasia with lower urinary tract symptoms (LUTS) \| \| Male stress incontinence \| \| Urinary obstruction \| \| Bladder outlet obstruction \| \| Benign prostatic hyperplasia \| \| BPH without urinary obstruction \| \| Overactive bladder \| \| Benign prostatic hyperplasia without lower urinary tract symptoms \| \| Bladder mass \| \| Suprapubic catheter (CMS/HCC) \| \| Prostate cancer metastatic to multiple sites (CMS/HCC) \| \| Status post implantation of artificial urinary sphincter \| \| Cystitis \| \| High grade prostatic intraepithelial neoplasia \| \| Atypical small acinar proliferation of prostate \| \| Lower urinary tract symptoms \| \| Transitional cell carcinoma, bladder (CMS/HCC) \| \| History of recurrent UTIs \| \| Recurrent cystitis \| \| S/P ileal conduit (CMS/HCC) \| \| Benign prostatic hypertrophy \| \| Abnormal prostate biopsy \| \| History of external beam radiation therapy \| \| Neoplasm of prostate, distant metastasis staging category M1a: metastasis to nonregional lymph nodes (CMS/HCC) \| \| BPH with elevated PSA \| \| History of prostate surgery \| \| Benign prostatic hyperplasia with urinary retention \| \| Prostate nodule \| \| Local recurrence of prostate cancer (CMS/HCC) \| \| Prostate cancer metastatic to intraabdominal lymph node (CMS/HCC) \| \| SUI (stress urinary incontinence), male \| \| Acute cystitis without hematuria \| \| Benign non-nodular prostatic hyperplasia with lower urinary tract symptoms \| \| Foley catheter in place \| \| Postoperative urinary retention \| \| Prostatism \| \| Effects of radiation \| \| Increased prostate specific antigen (PSA) velocity \| \| Bladder stone \| \| Granulomatous cystitis \| \| History of therapeutic radiation \| \| Radiation \| \| Erectile dysfunction after radical prostatectomy \| \| S/P prostatectomy \| \| Lesion of bladder \| \| Metastatic castration-resistant adenocarcinoma of prostate (CMS/HCC) \| \| Effects of radiation, unspecified \| \| Recurrent prostate cancer (CMS/HCC) \| \| OAB (overactive bladder) \| \| Primary prostate adenocarcinoma (CMS/HCC) \| \| Erectile dysfunction following radical prostatectomy \| \| H/O therapeutic radiation \| \| Retention of urine \| \| Ileostomy in place (CMS/HCC) \| \| Urothelial cancer (CMS/HCC) \| \| Benign prostatic hyperplasia with nocturia \| \| Urge incontinence \| \| CIS (carcinoma in situ of bladder) \| \| Attention to ileostomy (CMS/HCC) \| \| CHEK2-related prostate cancer (CMS/HCC) \| \| Prostate cancer metastatic to pelvis (CMS/HCC) \| \| Stress incontinence (female) (male) \| \| Spastic bladder \| \| Benign prostatic hyperplasia with post-void dribbling \| \| Personal history of prostate cancer \| \| Urinary bladder stone \| \| Bladder tumor \| \| Interstitial cystitis \| \| Benign prostatic hyperplasia with weak urinary stream \| \| PIN III (prostatic intraepithelial neoplasm III) \| \| Acquired bladder neck obstruction \| \| Incontinence \| \| Incontinence of urine \| \| Chronic prostatitis \| \| Urothelial carcinoma of bladder (CMS/HCC) \| \| Adenocarcinoma of prostate (CMS/HCC) \| \| Prostatic enlargement \| \| Urinary incontinence, mixed \| \| Carcinoma of prostate (CMS/HCC) \| \| Malignant neoplasm of lateral wall of urinary bladder (CMS/HCC) \| \| Mixed stress and urge urinary incontinence \| \| PIN (prostatic intraepithelial neoplasia) \| \| Prostatitis, acute \| \| Stress incontinence, male \| \| Benign prostatic hyperplasia with urinary hesitancy \| \| Urinary incontinence, urge \| \| Acute prostatitis \| \| Metastasis from hormone-refractory prostate cancer (CMS/HCC) \| \| Abnormal prostate exam \| \| H/O prostatectomy \| \| Malignant neoplasm of urinary bladder (CMS/HCC) \| \| History of radiation exposure \| \| Malignant tumor of prostate (CMS/HCC) \| \| Nodular prostate without lower urinary tract symptoms \| \| Prostate cancer metastatic to intrapelvic lymph node (CMS/HCC) \| \| Prostate carcinoma (CMS/HCC) \| \| Urothelial carcinoma (CMS/HCC) \| \| Benign prostatic hyperplasia with urinary obstruction \| \| History of acute prostatitis \| \| History of prostatitis \| \| Cystitis with hematuria \| \| Prostate disease \| \| Unspecified urinary incontinence \| \| History of bladder cancer \| \| Urgency incontinence \| \| Bladder stones \| \| H/O prostatitis \| \| Hx of radiation therapy \| \| Benign hypertrophy of prostate \| \| Benign prostate hyperplasia \| \| Prostate nodule with urinary obstruction \| \| Bladder disorder \| \| Prostate hypertrophy \| \| Prostate neoplasm \| \| Ileal conduit stomal stenosis \| \| Primary bladder adenocarcinoma (CMS/HCC) \| \| **Endpoint: “Diagnosis everything” table** \| \| Elevated prostate specific antigen (PSA) \| \| Elevated prostate specific antigen \| \| **Endpoint: “Clarify Medical History” table** \| \| Elevated PSA \| \| Elevated prostate specific antigen (PSA) \| \| History of elevated PSA \| \| PSA elevation \| \| Raised prostate specific antigen \| \| Abnormal PSA \| \| Abnormal prostate specific antigen (PSA) \| \| Elevated PSA, between 10 and less than 20 ng/ml \| \| Abnormal prostate specific antigen test \| \| Rising PSA level \| \| Elevated PSA, less than 10 ng/ml \| |
| --- | --- | --- | --- | --- | --- | --- | --- | --- | --- | --- | --- | --- | --- | --- | --- | --- | --- | --- | --- | --- | --- | --- | --- | --- | --- | --- | --- | --- | --- | --- | --- | --- | --- | --- | --- | --- | --- | --- | --- | --- | --- | --- | --- | --- | --- | --- | --- | --- | --- | --- | --- | --- | --- | --- | --- | --- | --- | --- | --- | --- | --- | --- | --- | --- | --- | --- | --- | --- | --- | --- | --- | --- | --- | --- | --- | --- | --- | --- | --- | --- | --- | --- | --- | --- | --- | --- | --- | --- | --- | --- | --- | --- | --- | --- | --- | --- | --- | --- | --- | --- | --- | --- | --- | --- | --- | --- | --- | --- | --- | --- | --- | --- | --- | --- | --- | --- | --- | --- | --- | --- | --- | --- | --- | --- | --- | --- | --- | --- | --- | --- | --- | --- | --- | --- | --- | --- | --- | --- | --- | --- | --- | --- | --- | --- | --- | --- | --- | --- | --- | --- | --- | --- | --- | --- | --- | --- | --- | --- | --- | --- | --- | --- | --- | --- | --- | --- | --- | --- | --- | --- | --- | --- | --- | --- | --- | --- | --- | --- | --- | --- | --- | --- | --- | --- | --- | --- | --- | --- | --- | --- | --- | --- | --- | --- | --- | --- | --- | --- | --- | --- | --- | --- | --- | --- | --- | --- | --- | --- | --- | --- | --- | --- | --- | --- | --- | --- | --- | --- | --- | --- | --- | --- | --- | --- | --- | --- | --- | --- | --- | --- | --- | --- | --- | --- | --- | --- | --- | --- | --- | --- | --- | --- | --- | --- | --- | --- | --- | --- | --- | --- | --- | --- | --- | --- | --- | --- | --- | --- | --- | --- | --- | --- | --- | --- | --- | --- | --- | --- | --- | --- | --- | --- | --- | --- | --- | --- | --- | --- | --- | --- | --- | --- | --- | --- | --- | --- | --- | --- | --- | --- | --- | --- | --- | --- | --- | --- | --- | --- | --- | --- | --- | --- | --- | --- | --- | --- | --- | --- | --- | --- | --- | --- | --- |

**PSA Gap**

PSA test results are reported alongside a reference range, which varies by age as follows:

- - 40-49yo: 0-2.5 ng/dL
  - 50-59yo: 0-3.5 ng/dL
  - 60-69yo: 0-4.5 ng/dL
  - >= 70yo: 0-6.5 ng/dl

PSA test results that exceed the upper limit of normal are flagged as “abnormal.”

PSA gap was calculated as the difference between a given PSA lab value and its corresponding upper limit of normal. For example, a 55-year-old male with a PSA value of 3.2 ng/dL would have a PSA gap of (3.2 – 3.5) = -0.3 and would be labelled “normal.” A 65-year-old with a PSA value of 5.2 ng/dL would have a PSA gap of (5.2 – 4.5) = +0.7 and would be labelled “abnormal.”

**Statistical Analysis (equation)**

Discontinuity at the PSA ULN (“normal”/”abnormal” cutoff) was estimated via logistic regression under the following formula, where p = probability of endpoint:

1. Log (p/(1-p)) = Intercept + β_1_ * Age + β_2_ * PSA gap + β_3_ * I(PSA gap >0) + β_4_ * PSA gap: I(PSA gap >0)

Where I() is the indicator function. Therefore, β_3_ describes the effect of the “abnormal” (versus “normal) flag on log odds of endpoint, after adjusting for age, PSA gap, and the relationship between PSA gap and PSA gap when “abnormal.” A statistically significant β_3_ rejects the null hypothesis of no discontinuity at the PSA ULN. Age was included as a term in this regression given its association with PSA level and a suspected association with study endpoints.

**Sensitivity Analysis**

As a sensitivity analysis, a similar approach was taken to age-designated subgroups with PSA modeled directly as the continuous variable rather than calculated PSA gap. Subgroups comprised, separately, patients aged 50-59 and 60-69 years old at the time of a screening PSA test.

The relevant formula is similar to that shown in the main manuscript. For example, amongst patients aged 50-59 the formula is as follows, where p is probability of endpoint (referral, early repeat PSA, MRI or biopsy [or both]):

1. Log (p/(1-p)) = Intercept + β_1_ * Age + β_2_ * PSA + β_3_ * (PSA >3.5) + β_4_ * (PSA:PSA >3.5)

Such that β_3_ describes the effect of the “abnormal” (versus “normal) flag on odds of endpoint, after adjusting for age, PSA, and the relationship between PSA and PSA when “abnormal.”

Again, age was included as term in this regression given its known association with PSA level and a suspected association with study endpoints.

A further sensitivity analysis modeled discontinuity of MRI or biopsy (or both) at the PSA ULN (via PSA Gap, equation 1 above), stratifying by date of PSA test. This probes the influence of possible changes in the downstream diagnostic pathway that have been adopted in recent years on the observed effect of the “abnormal flag.”

**Supplementary Figure 1.** Baseline characteristics as a function of calculated PSA gap: (a) age, (b) PSA value, (c) proportion of patients of African ancestry, (d) proportion of patients with family history of prostate cancer. Error bars represent 95% confidence interval.

**Supplementary Figure 2.** Proportion of PSA tests amongst patients aged 50-59 followed by (a) referral to urologist within six months, (b) repeat PSA test within six months, and (c) receipt of either prostate MRI or prostate biopsy (or both) within six months; by PSA (ng/dL). Pink shading corresponds to PSA tests labelled as “abnormal.”

**Supplementary Figure 3.** Proportion of PSA tests amongst patients aged 60-69 followed by (a) referral to urologist within six months, (b) repeat PSA test within six months, and (c) receipt of either prostate MRI or prostate biopsy (or both) within six months; by PSA (ng/dL). Pink shading corresponds to PSA tests labelled as “abnormal.”

**Supplementary Table 2.** Multivariable models assessing regression discontinuity of (a) referral, (b) early repeat PSA, and (c) MRI or biopsy (or both) amongst patients aged 50-59 at the PSA ULN (3.5 ng/dL for this age group) .

(a)

|  | Odds Ratio | 95% CI Lower Bound | 95% CI Upper Bound | P-Value |
| --- | --- | --- | --- | --- |
| Age (per 1 year) | 0.989565 | 0.930632 | 1.052703 | 0.73 |
| PSA | 6.205301 | 5.43978 | 7.142274 | <0.001 |
| PSA >3.5 ng/dL | 18.17073 | 8.227195 | 39.8009 | <0.001 |
| Interaction of PSA with PSA >3.5 ng/dL | 0.163895 | 0.141272 | 0.189749 | <0.001 |

(b)

|  | Odds Ratio | 95% CI Lower Bound | 95% CI Upper Bound | P-Value |
| --- | --- | --- | --- | --- |
| Age (per 1 year) | 1.012539 | 0.979653 | 1.046719 | 0.46 |
| PSA | 2.760219 | 2.582232 | 2.953461 | <0.001 |
| PSA >3.5 ng/dL | 4.654083 | 2.235214 | 9.307127 | <0.001 |
| Interaction of PSA with PSA >3.5 ng/dL | 0.371846 | 0.343083 | 0.40786 | <0.001 |

(c)

|  | Odds Ratio | 95% CI Lower Bound | 95% CI Upper Bound | P-Value |
| --- | --- | --- | --- | --- |
| Age (per 1 year) | 0.976582 | 0.910872 | 1.04773 | 0.50 |
| PSA | 3.920311 | 3.484698 | 4.437337 | <0.001 |
| PSA >3.5 ng/dL | 8.788608 | 3.788182 | 18.20651 | <0.001 |
| Interaction of PSA with PSA >3.5 ng/dL | 0.267348 | 0.233759 | 0.30697 | <0.001 |

**Supplementary Table 3.** Multivariable models assessing regression discontinuity of (a) referral, (b) early repeat PSA, and (c) MRI or biopsy (or both) amongst patients aged 60-69 at the PSA ULN (4.5 ng/dL for this age group) .

(a)

|  | Odds Ratio | 95% CI Lower Bound | 95% CI Upper Bound | P-Value |
| --- | --- | --- | --- | --- |
| Age (per 1 year) | 0.939178 | 0.887454 | 0.993306 | 0.028 |
| PSA | 3.531493 | 3.221229 | 3.891878 | <0.001 |
| PSA >4.5 ng/dL | 24.80956 | 4.747749 | 94.14171 | <0.001 |
| Interaction of PSA with PSA >4.5 ng/dL | 0.29934 | 0.261387 | 0.365819 | <0.001 |

(b)

|  | Odds Ratio | 95% CI Lower Bound | 95% CI Upper Bound | P-Value |
| --- | --- | --- | --- | --- |
| Age (per 1 year) | 1.003514 | 0.973685 | 1.034157 | 0.82 |
| PSA | 1.972732 | 1.878396 | 2.073086 | <0.001 |
| PSA >4.5 ng/dL | 2.146618 | 0.314307 | 9.576111 | 0.37 |
| Interaction of PSA with PSA >4.5 ng/dL | 0.607458 | 0.519043 | 0.778204 | <0.001 |

(c)

|  | Odds Ratio | 95% CI Lower Bound | 95% CI Upper Bound | P-Value |
| --- | --- | --- | --- | --- |
| Age (per 1 year) | 1.004708 | 0.932185 | 1.082765 | 0.90 |
| PSA | 2.738304 | 2.481899 | 3.037981 | <0.001 |
| PSA >4.5 ng/dL | 23.25519 | 6.995415 | 66.83577 | <0.001 |
| Interaction of PSA with PSA >4.5 ng/dL | 0.39236 | 0.343678 | 0.454659 | <0.001 |

**Supplementary Table 4.** Multivariable models assessing discontinuity of MRI or biopsy (or both) at the PSA ULN (PSA Gap > 0) during (a) July 2015 – June 2019 (b) July 2019 – July 2023.

(a)

|  | Odds Ratio | 95% CI Lower Bound | 95% CI Upper Bound | P-Value |
| --- | --- | --- | --- | --- |
| Age (per 1 year) | 1.02165051 | 1.00238639 | 1.04164789 | 0.03 |
| PSA Gap | 2.37603693 | 1.81274402 | 3.13644241 | <0.001 |
| PSA Gap > 0 | 17.6005333 | 9.88723433 | 32.9820642 | <0.001 |
| Interaction of PSA Gap with PSA Gap > 0 | 0.42391056 | 0.32105229 | 0.55584185 | <0.001 |

(b)

|  | Odds Ratio | 95% CI Lower Bound | 95% CI Upper Bound | P-Value |
| --- | --- | --- | --- | --- |
| Age (per 1 year) | 1.01343945 | 0.99091801 | 1.03670717 | 0.2 |
| PSA Gap | 3.04767129 | 2.23743565 | 4.19474397 | <0.001 |
| PSA Gap > 0 | 9.55929762 | 4.98866043 | 19.4536239 | <0.001 |
| Interaction of PSA Gap with PSA Gap > 0 | 0.34304895 | 0.24891526 | 0.46806098 | <0.001 |
